# Major Chromosomal Breakpoint Intervals in Breast Cancer Co-Localize with Differentially Methylated Regions

**DOI:** 10.3389/fonc.2012.00197

**Published:** 2012-12-27

**Authors:** Man-Hung Eric Tang, Vinay Varadan, Sitharthan Kamalakaran, Michael Q. Zhang, Nevenka Dimitrova, James Hicks

**Affiliations:** ^1^Cold Spring Harbor LaboratoryCold Spring Harbor, NY, USA; ^2^Department of Oncology, Clinical Sciences, Lund UniversityLund, Sweden; ^3^Philips Research North AmericaBriarcliff Manor, NY, USA; ^4^The University of Texas at DallasRichardson, TX, USA; ^5^Tsinghua UniversityBeijing, China

**Keywords:** DNA methylation, copy number variation, Alu repeat element, genome instability, multi-modal analysis, breast cancer

## Abstract

Solid tumors exhibit chromosomal rearrangements resulting in gain or loss of multiple chromosomal loci (copy number variation, or CNV), and translocations that occasionally result in the creation of novel chimeric genes. In the case of breast cancer, although most individual tumors each have unique CNV landscape, the breakpoints, as measured over large datasets, appear to be non-randomly distributed in the genome. Breakpoints show a significant regional concentration at genomic loci spanning perhaps several megabases. The proximal cause of these breakpoint concentrations is a subject of speculation, but is, as yet, largely unknown. To shed light on this issue, we have performed a bio-statistical analysis on our previously published data for a set of 119 breast tumors and normal controls (Wiedswang et al., 2003), where each sample has both high-resolution CNV and methylation data. The method examined the distribution of closeness of breakpoint regions with differentially methylated regions (DMR), coupled with additional genomic parameters, such as repeat elements and designated “fragile sites” in the reference genome. Through this analysis, we have identified a set of 93 regional loci called breakpoint enriched DMR (BEDMRs) characterized by altered DNA methylation in cancer compared to normal cells that are associated with frequent breakpoint concentrations within a distance of 1 Mb. BEDMR loci are further associated with local hypomethylation (66%), concentrations of the Alu SINE repeats within 3 Mb (35% of the cases), and tend to occur near a number of cancer related genes such as the protocadherins, AKT1, DUB3, GAB2. Furthermore, BEDMRs seem to deregulate members of the histone gene family and chromatin remodeling factors, e.g., JMJD1B, which might affect the chromatin structure and disrupt coordinate signaling and repair. From this analysis we propose that preference for chromosomal breakpoints is related to genome structure coupled with alterations in DNA methylation and hence, chromatin structure, associated with tumorigenesis.

## Introduction

Breast cancer is a complex disease characterized by a combination of multiple genetic and epigenetic changes that have been widely studied in the past two decades. Pioneering works by Perou et al. (2000) and Sørlie et al. (2003) showed that breast cancer tumors consist of five expression-based molecular subtypes with different clinical outcomes. Genome instability in breast cancer has also been extensively characterized, first using array CGH based methods such as in Hicks et al. (2006), Kamalakaran et al. (2009), Bergamaschi et al. (2006), Chin et al. (2006), André et al. (2009), and more recently using high-resolution 500 k SNP arrays in, for example Haverty et al. (2008). These studies showed that cancer genomes are highly unstable, with recurrent, subtype specific rearrangements, defining groups that are consistent with existing molecular subtypes (Weigman et al., 2011). Furthermore, genome rearrangements occur in a non-random manner with copy number gains in 1q, 8q11, 11q, 17q, 20q, and losses in 5q, 6q, and 8p. These regions harbor cancer related genes such as TP53, CDKN2A, ERBB2, KRAS, PTEN, and are therefore extensively cataloged. In Hicks et al. (2006), three patterns were defined to qualitatively classify genome rearrangement profiles of breast tumors. A formalization of the model was proposed recently in Russnes et al. (2010), using scores to quantify the complexity of genome-wide architectural distortion. They have proposed that these patterns of genomic architecture could be used as prognostic markers.

In addition to large scale rearrangements of DNA, the characterization of cancer methylomes and their corresponding normal profiles has demonstrated that cancer genomes also undergo a remarkable amount of epigenetic disruptions leading to activation and silencing of genes involved in cancer related pathways. For example, the BRCA1 gene promoter is often hyper-methylated in hereditary breast cancers (Tapia et al., 2008). Furthermore, studies such as Kamalakaran et al. (2010) showed that Luminal and non-Luminal breast cancer tumors have different methylation patterns and that differentially methylated genes are associated with relapse risk and overall survival. More recently, using a cohort of 187 normal/breast cancer paired samples, a study showed that subtype specific changes in DNA methylation are associated with expression-based subtypes Luminal A, B, HER2 positive, and basal-like tumors (Bediaga et al., 2010). Similarly, the analysis of cancer related genes in fresh frozen breast tumor samples showed that Luminal A, B and basal-like tumors had distinct methylation patterns, with a higher methylation frequency in the Luminal B and a lower frequency in the basal-like subtype (Holm et al., 2010).

These studies point to a probable existence of mechanistic cross-talk between epigenetic modifications, genome instability, and transcriptional programs within breast cancers.

While a majority of studies follow a mono-modal approach, multi-modal analysis seem to be more suited to characterize this complex disease involving such diverse molecular, genetic, epigenetic factors. Combining DNA methylation and gene expression profiles of cancer tissues has shown a strong inverse correlation between gene expression and promoter methylation levels (Kamalakaran et al., 2010). Furthermore, the relation between copy number variation (CNV) and gene expression was similarly studied, looking at the impact of the chance of DNA copies of each gene on their expression, and looking for potential therapeutic targets (Staaf et al., 2010). Multi-modal platforms have been designed to identify complex signatures of breast cancer. For example, ER status has been correlated with differences in methylation, expression, and DNA copy number (Sun et al., 2011). More importantly, in the recent large scale integrated analysis of 2000 breast tumors (Curtis et al., 2012), novel molecular subgroups were defined based on the inter-relationship between inherited genomic variants, somatic copy number alterations and their impact on the transcriptome.

Although gene-centered, these methodologies are important in defining predictive or prognostic signatures, or common aberrations, in each type of cancer. However, these methodologies provide little insight into the mechanisms that drive these epigenetic and genetic changes on a genome-wide scale. To begin to probe these mechanisms we have re-examined published data in order to look for relationships between epigenetic gene regulation and the physical alterations associated with cancer. We ask several questions: (1) What is the relationship between the breakpoints in chromosomal rearrangements and DNA methylation? (2) If correlated, what is the overlap between these differentially methylated breakpoints with regions in the genome that are prominently deregulated in cancer? (3) What is the relationship between breakpoint dense and differentially methylated regions (DMR) and repetitive elements across the genome? To address the problem, we developed a model integrating a combination of statistical and experimental methods. Genome-wide profiling of DNA methylation and DNA copy number was performed on 108 tumor and 11 adjacent normal tissue samples from a Norwegian breast cancer cohort (Wiedswang et al., 2003) using in-house analysis platforms: Methylation Oligonucleotide Microarray Analysis (MOMA; Kamalakaran et al., 2009) and Representational Oligonucleotide Microarray Analysis (ROMA; Lucito et al., 2003). Systematic identification of DMRs and Alu enriched loci was performed with regards to major genome rearrangements and breakpoint enriched regions (BERs).

Our work uncovers several lines of evidence relating major genome rearrangements and breakpoint rich regions, with differential methylation patterns, local repeat enrichment, and functional enrichment in these regions. These different observations will allow us to understand better the mechanisms underlying rearrangement events in breast cancer and their relation to the other molecular and epigenetic anomalies.

## Materials and Methods

### Tumor sample set

We used the 119 Norwegian breast cancer dataset (the Oslo Metastases Study) described in Wiedswang et al. (2003), Naume et al. (2007), and Russnes et al. (2010). These samples were part of the cohort that established molecular subtypes. The subtypes were established by the original study, by using the correlation to the expression centroids of the intrinsic genes from microarray expression data, described in Sørlie et al. (2003).

Each patient of the study is further classified into one of the following subgroups: luminal A tumor subtype (40 patients), Luminal B (15), ERBB2 positive (19), basal-like (12), normal-like (14), and eight undefined. The normal tissue dataset consisted of 11 adjacent breast tissue samples. For each sample, DNA methylation and CNV analysis was performed. We used the DNA methylation MOMA analysis data previously published in Kamalakaran et al. (2010) and the copy number ROMA analysis data previously published in Hicks et al. (2006). The MOMA and ROMA experimental platforms are described below.

### ROMA platform

To measure CNV across the genome, we used the ROMA platform described in Lucito et al. (2003). The genome is covered by regularly spaced 82,055 probes printed on an array, providing a coverage of the genome at 40,000 nucleotides resolution. Copy number ratios are measured using the skin fibroblast CHPSKN-1 cell-line as reference. Since CHPSKN-1 cells come from a male individual, we focused our analysis on the 22 autosomes only. Multiple segmentation schemes were used in the development of the ROMA platform to obtain copy number values, before settling on the Circular Binary Segmentation (CBS) algorithm (Venkatraman and Olshen, 2007). This scheme requires three consecutive probes to define a change in copy number value. A whole genome comparison of the ROMA platform with the Agilent 44 k and Illumina 109 k aCGH platforms showed overall similarity with minor differences in amplitude and number of events (Baumbusch et al., 2008), and FISH probes were used to validate the copy number calls in the “firestorm” regions (Hicks et al., 2006).

### CNV analysis across tumor samples

We partitioned the genome into variable windows such that each sample is observed in a single segmented copy number state (amplified, deleted, normal copy number). Windows are determined by all the breakpoints obtained by segmentation of the copy number values in each sample using the CBS algorithm. Longer intervals describe regions that have very little copy number change across all the patients while short intervals correspond to regions with high copy number changes, i.e., many breaks across different samples.

We defined three levels of amplification in order to bin samples into three categories. In each given interval, samples with a ROMA ratio greater than 1.1 are defined as amplified, samples with ratio less than 0.9 are defined as deleted, and if their ROMA ratio fall between these two values are defined as normal. The thresholds that define the normal copy number ratio were chosen empirically to take into account the measurement noise around 1. The CNV profile of the dataset can be then plotted as the fraction of sample showing amplifications and deletions across.

### Breakpoint enriched region detection

We used the segment’s start and end defined by the CBS algorithm for the CNV profile of each sample to define our breakpoints. We then calculated the density function using the R function with a bandwidth of 1 Mb and defined the center of the breakpoint dense region as the local maxima of the density.

### MOMA platform

We surveyed the methylome of each tumor sample using the MOMA platform (Kamalakaran et al., 2009). Each CpG island is covered by one or several MOMA fragments that undergo *Msp*I cleavage and McrBC or mock digestion. McrBC and mock digested fragments are then labeled and hybridized on a chip. The hybridization ratio reflects the level of methylation of the probed CpG island. In total, the 27,000 CpG islands annotated by the UCSC genome browser (hg17 build) are covered by 159,436 MOMA fragments. The data is normalized by converting the hybridization log-ratios into the probabilistic space using an Expectation-Maximization (EM) method (Kamalakaran et al., 2010; and Supplementary text). Each MOMA fragment is assigned one of the following states: high methylation (+1), low methylation (−1), and 0 state for cases falling in none of the two categories.

### Differentially methylated region detection

To identify local variations of DNA methylation in the 108 breast cancer samples, we compared the distribution of methylations calls within each of the intervals defined by all the copy number breakpoints with the one observed across the genome. Each MOMA fragment is surveyed and we can associate to each fragment a triplet of observations accounting for the number of “+1,” “0,” and “−1”s seen across all samples. For example, a window can be seen 30 times as “+1,” 3 times as “0,” and 7 times as “−1.”

To identify local changes DNA methylation across the genome, we use the Hotelling’s *T*^2^-test, a generalization the Student’s *T*-test for multivariate hypothesis testing. The null hypothesis *H*_0_ is defined as the observed distribution of “+1,” “0,” and “−1”s observed at each fragment across the MOMA platform. It is calculated based on 159436 observations. It has an expectation μ*_0_* = *(*μ_01_, μ_02_, μ_03_) and covariance *B*. If a window contains n MOMA fragments, let *X*_1_, *X*_2_, …, *X_n_* be *n* independent three-dimensional vectors, *n* *−* 1 ≥ 3. *X*_1_, *X*_2_, …, *X_n_* follows the normal law *N*(μ,*B*). Then, the *T*^2^ statistic can be expressed as:

(1) $$T^{2}=n{\left(\mu_{\text{X}}-\mu_{0}\right)}^{T}S^{-1}\left(\mu_{X}-\mu_{0}\right)$$

where

(2) $$\mu_{x}=\frac{1}{n}\overset{n}{\underset{i=1}{\sum }}X_{i}$$

and

(3) $$S^{-1}=\frac{1}{n-1}\overset{n}{\underset{i=1}{\sum }}\left(X_{i}-\mu_{x}\right){\left(X_{i}-\mu_{X}\right)}^{t}$$

are the sample maximum likelihood estimators of μ and *B*. Then *T^2^* has the Hotelling’s *T*-square distribution and the statistic

(4) $$F=\frac{n-p}{p\left(n-1\right)}T^{2}$$

has a Fisher’s *F* distribution with *p* and *n − p* degrees of freedom, *p* = 3 and parameter (μ − μ_0_)*^T^B*^−1^(μ − μ_0_)${\left(\mu -\mu_{0}\right)}^{T}B^{-1}\left(\mu -\mu_{0}\right)$.

To test whether the null hypothesis *H*_0_:μ = μ_0_ is rejected, we compute the *F* statistics using the observations *X*_1_, *X*_2_, …, *X_n_* of the three-dimensional normal law *N*(μ, *B*) and derive the associated *p*-value. We then perform a Benjamini and Hochberg (1995) False discovery Rate (FDR) correction on the obtained statistics. A window is considered to have significant deviation in its methylation pattern if its *p*-value is smaller than 10^−2^.

### Breakpoint enriched differentially methylated region detection

To detect association between BER and DMR we measured the cumulative number of DMRs as a function of the distance to the nearest BER (see Figure S1 in Supplementary Material) and compared the distance distributions of the observed occurrences in tumor with randomized locations derived using a null model. To choose the most suitable null model, we first plotted the distribution of distances between two DMRs, shown in Figure S2 in Supplementary Material). We evaluated three different null models (uniform, normal, and gamma model) with differing degrees of similarity to the observed distribution of distances between locations of methylation deviation. The shape of each null model compared to that of the observed data is shown in the Figure S3 in Supplementary Material The uniform distribution is least similar to the observed distribution and thus the least stringent of null models, as compared to observed the normal distribution is somewhat more stringent and finally the gamma distribution is the most realistic null model and thus the most stringent null model. Then we compute the mean cumulative distributions of the randomized locations based on the individual null models (after 1000 randomizations). In addition, we carried out an FDR-corrected Wilcoxon test to compare the observed distribution with the one generated by the gamma as the null model. This test was designed to identify the locations of maximal difference between the observed and null model curves.

### Repeat enriched loci detection

To identify local changes of Alu repeat frequencies, we used the Repeat masker database (hg17) as reference and compared the repeat enrichment frequencies in our regions of interest with the one observed across the genome. An FDR-corrected Wilcoxon test was performed for each repeat type (AluJ, AluS, AluY), in every non-overlapping sliding window of 100 kb. An empirical threshold of *p* < 0.001 was used to decide whether the tested region was significantly enriched or not.

## Results

**Figure 1:**
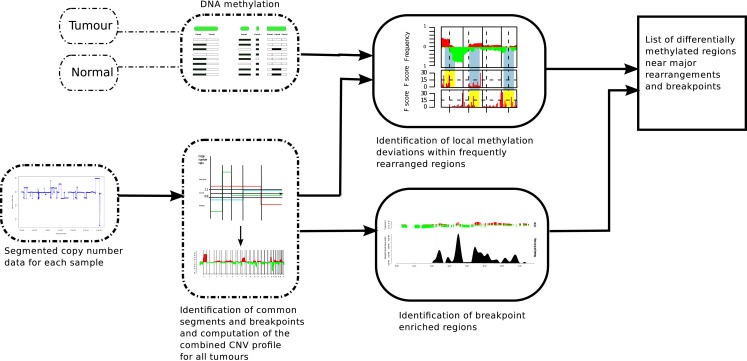
**Analysis method in order to find DMRs associated with BERs**. ROMA genome-wide copy number profiles from breast tumors were combined to partition the genome into variable intervals of stable copy number state in which we estimate DNA methylation levels using MOMA measurements from tumor and Normal samples. A Hotelling’s *T*^2^-test is performed to identify significant DMRs. On the other track, the locations of BERs are obtained from the ROMA profiles and the list of both significant DMRs and BERs are further evaluated for statistical association (Figure 3).

The model shown in Figure 1 is conceptualized to integrate copy number and DNA methylation patterns in order to determine if there is a mechanistic association between the location of major chromosomal breakpoints and local DNA methylation changes. In order to address this question, we first need to define the genomic regions in which the associations can be tested. Using the ROMA genome-wide copy number profiles of the 108 breast tumors, we partition the genome into variable intervals, delineated by the density of breakpoint observations, so called BER. Next, we identified frequently differentially methylated regions (DMR) in tumor samples compared to normal samples (using MOMA and Hotelling’s *T*^2^-test, BH *p* < 0.01, see Materials and Methods).

**Figure 2:**
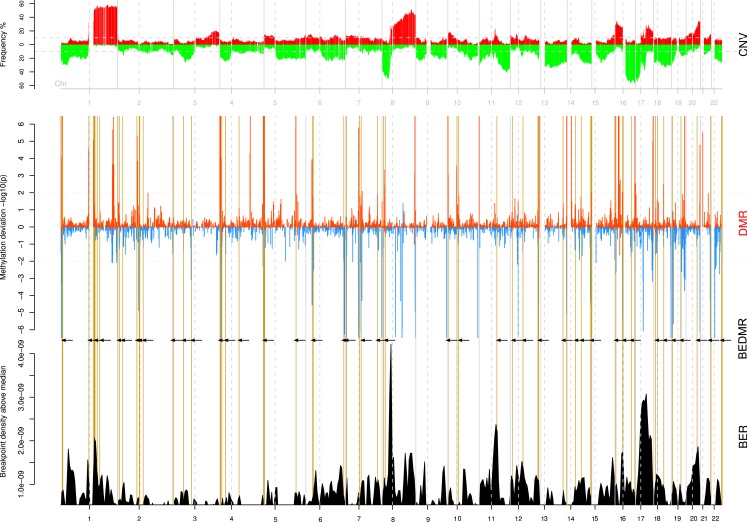
**Significant DMRs tend to co-localize with breakpoints enriched regions**. The copy number profile of all 108 breast tumors is shown on the top track (CNV). The middle track (DMR) shows the amplitude of the DNA methylation level change compared to normal across genome. Hypo-methylated regions are assigned a negative score, defined as log10(*p*) while hyper-methylated regions take a score equal to −log(*p*). Significant DMRs are marked by peaks with a score greater than ±2. The bottom track (BER) shows the locations of breakpoint enriched regions. Breakpoint enriched DMRs (BEDMR), i.e., DMRs occurring in the vicinity of a BER are marked by vertical yellow lines and black arrows.

It was interesting to see that DMRs were spread across the genome and out of the 217 DMRs, 145 were hypo-methylated, and 72 were hyper-methylated loci compared to the normal. In Figure 2, we summarize the integrative analysis on the whole genome. The top track CNV recapitulates the copy number gains and losses as frequencies among all the tumor samples. The scores and locations of significant DMR are shown in the middle track (DMR). A positive score means that the surveyed window is hyper-methylated compared to the baseline for normal samples while a negative score indicates a local hypomethylation. We identified 217 DMR regions in all tumor samples (BH *p* < 0.01). Finally, we combine these DMR with the BER, shown in the bottom track (BER) in order to test their associations. To guide the reader across the different tracks, we highlight breakpoint enriched DMR (BEDMR) with vertical yellow lines that visually link DMRs that appear to co-localize with BERs, within a distance of 1 Mb (arrows inserted to accentuate locations of BEDMRs). In the next section, we provide a more objective measure of this association.

### Significant DMRs in tumors co-localize with breakpoints

**Figure 3:**
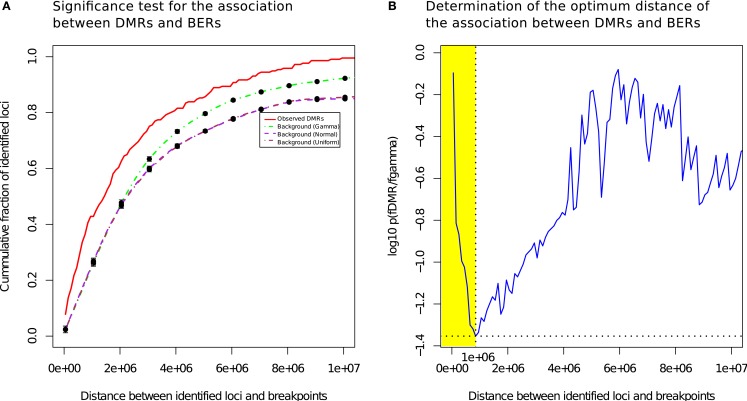
**Differentially methylated regions co-localize with breakpoints enriched regions**. **(A)** DMRs tend to be more proximal to BER than expected **(B)**: the most significant distance of the association between DMRs and BERs occurs at a distance of 1 Mb (shown in yellow).

We measured the cumulative number of DMRs as a function of the distance to the nearest BER and compared the distance distributions of the observed occurrences in tumor with randomized locations derived using a null model (see Materials and Methods). Figure 3A presents the mean cumulative distributions of the randomized locations based on the individual null models alongside with the observed data.

The cumulative frequency curve obtained with the dataset of all tumor samples (red) shows that DMR occur more frequently than random events generated by the normal (purple) and uniform (brown) or gamma (green) models in the 0–10 Mb distance range away from a BER, suggesting the existence of a positional bias (Wilcoxon test, FDR-corrected *p* < 0.05; Figure 3B).

The best *p-*value score (*p* = 0.039), i.e., the lowest value on the blue curve, was reached at a distance of 1 Mb, where 42.8% of the observed DMR were found (73% occur within 2 Mb). In contrast, only 23.7% of the regions in the gamma null model are within 1 Mb of BERs. In addition, changing null models did not affect dramatically the result, showing that the DNA methylation change events that we found are consistently co-occurring near breakpoints rich regions in a non-random manner. We also observed that the co-localization of DMR and BERs is more significant than the expectation in each individual subtype and irrespective of each subtype data was used (see Figure S4 in Supplementary Material).

To summarize, we found that 93 of the identified 217 DMRs in our set of 108 breast tumors compared to 11 normal samples significantly co-occurred with BERs within a distance of 1 Mb (Detailed summary is presented in Figure S6 in Supplementary Material and Table S1 in Supplementary Material). This result provides the evidence of a likely association between differentially methylated and BERs within a distance of 1 Mb (shaded yellow in Figure 3B). In the following, we will use the shorthand BEDMR (breakpoint enriched DMR) to designate these regions.

**Figure 4:**
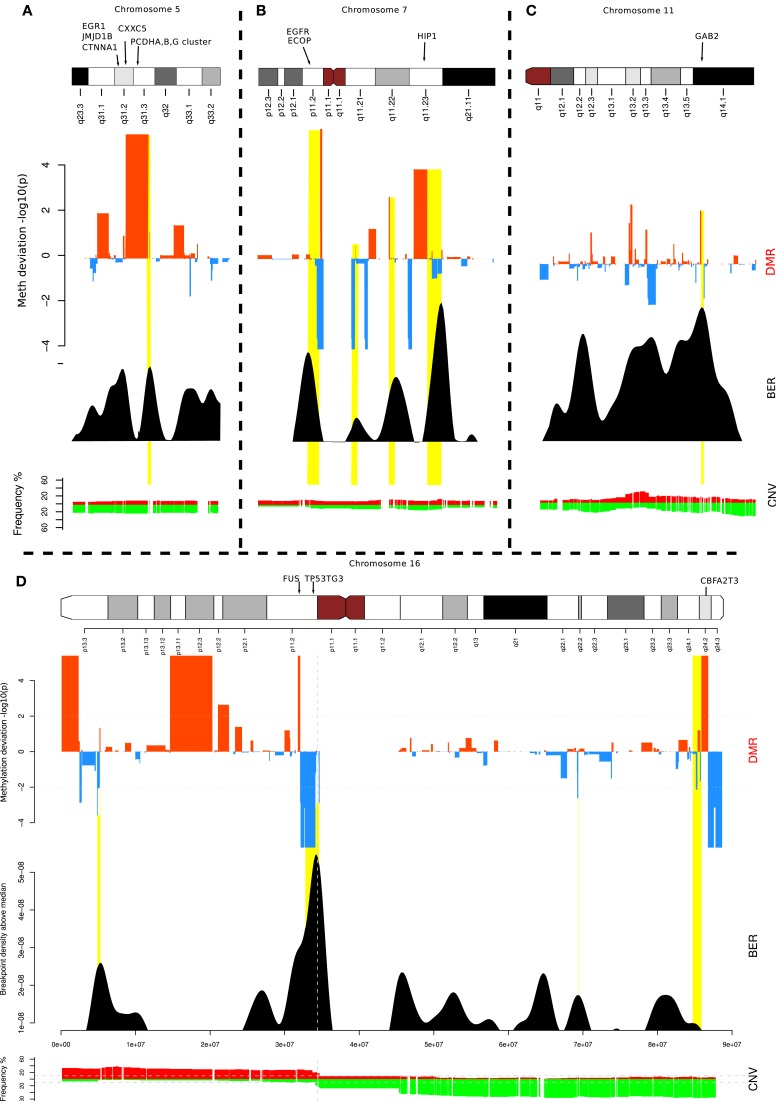
**Localization of BEDMRs in the genome**. BEDMRs tend to occur in genomic contexts. For example **(A)** 5q31.3 (PCDHA,B,G cluster) **(B)** 7p11.2 and 7q11.23 (EGFR, HIP1) **(C)** 11q14.1 (GAB2). **(D)** 16p13.3, 16p11.2, 16q24.2 (TSC2, FUS, P53TG3, CBFA2T3). These regions contain important cancer related genes and can be both deleted and hyper-methylated **(A)** or amplified and demethylated **(C)**.

To investigate further, we focused on a few loci located on chromosomes 5, 7, 16, and 11 (Figure 4). These examples illustrate the different contexts in which we find BEDMRs: intra-chromosomal (Figures 4A,C), and peri-centromeric (Figures 4B,D), involving whole-arm rearrangement events. We found that BEDMRs were ubiquitous and not biased toward repetitive regions such as telomeres or centromeres.

We analyzed the gene content of the 93 identified BEDMRs (Table S2 in Supplementary Material) and reported remarkable copy number and methylation status in at least 20% of the patients (Table S3 in Supplementary Material). The annotation was obtained using the ROMA/MOMA data and we reported all the genes in the vicinity of a MOMA fragment. We found that 71 regions contained genes.

When looking at the genes contained in the BEDMRs, we found that many of the discovered regions have been previously linked with breast cancer and contained known oncogenes. In Figure 4A, we highlight the gene-dense 5q31.2 locus. It contains the protocadherin gene cluster, PCDHA, B and G and genes involved in cancer such as EGR1, CTNN1A, JMJD1B, and CXXC5. It has been shown that this locus was subject to agglomerative epigenetic aberration and reported to be epigenetically silenced in cancer (Novak et al., 2008). In Figure 4B, we show the peri-centrometic region of chromosome 7, with centromere proximal and intra-chromosomal BEDMRs further down in the q-arm. The hypo-methylated and amplified BEDMR on 7p11.2 is functionally important since it is located about 1 Mb downstream of the locus containing EGFR. In addition we found HIP1, a regulator of EGFR in the endocytic pathway at the boundary of the hyper-methylated BEDMR in 7q11.23. Figure 4C shows the q-arm of chromosome 11, which undergoes intense copy number changes, in particular the hemizygous deletion of the GAB2 locus (11q14.1). The GAB2 gene, located within a methylated BEDMR, in unmethylated in 73% of the samples. This gene was reported to inhibit E-cadherin expression and to enhance the expression of ZEB1, a transcription factor involved in epithelial-to-mesenchymal transition and cell migration and invasion through the activation of the PI3K pathway (Wang et al., 2012). In our last example, Figure 4D, we showed the whole chromosome 16 which involves a whole-arm amplification (16p) and deletion event (16q). The peri-centromeric breakpoint is located near the locus containing the FUS oncogene and the unmethylated BEDMR containing TP53TG3.

In addition to these examples, we looked at databases of cancer related genes such as the Cancer Gene Census (Futreal et al., 2004), which reports a list of 487 genes with mutations that have been causally associated with cancer. We found that 8 of our 93 regions contained such types of genes, nine in total, including AKT1, ARNT, PMS2, and the oncogenic ubiquitin hydrolase, DUB3 for which we previously reported abnormal demethylation in our integrated study of ovarian cancer (Wrzeszczynski et al., 2011). Furthermore, we performed a manually curated literature search using the text-mining tool pubmatrix (Becker et al., 2003) to identify all the genes located within BEDMRs that have been previously linked with cancer. We found that 57% of the regions had at least a gene with three matches. In total, 39% of the genes (244/623) in these regions had at least one match, and 29% (184/623/599) at least three matches in the literature. (Table S2 in Supplementary Material).

The described results provided lines of evidence that many BEDMRs were proximal to important cancer genes, although there was no strong positive selection from the statistics.

To investigate further the functional importance of the BEDMRs, we looked whether these genes were undergoing epigenetic and genetic regulatory processes. In Table S3 in Supplementary Material, we listed genes found in BEDMRs with a remarkable copy number and DNA methylation status in at least 20% of the patients. One could see that several of these loci undergo cumulative genetic and epigenetic regulatory effects, favoring either silencing or an increase of gene expression. For example, the 5q31.2 locus, containing the protocadherin gene family, EGR1, CTNN1A, JMJD1B, and CXXC5 is hyper-methylated and in decreased copy number in 22% of the patients. We also found that the histone gene cluster on 1q21.2 was hypo-methylated and amplified (in 34–52% of the samples for each gene), so was 6p22.1 which was in the 217 DMRs and not in the 93 BEDMR loci. Although we found many deleted and methylated BEDMRs such as the protocadherin cluster, a large majority of BEDMR loci were hypo-methylated compared to normal (61/93). For example, centromeric regions tend to be methylated, however, in Figures 4B,D, we found a local decrease of DNA methylation level. This can be associated with local structure remodeling allowing transcription (Wong et al., 2006). Actually, the region in chromosome 7 (see Figure 4B) shows both peri-centromeric and intra-chromosomal BEDMRs. The observed bias toward amplification and demethylation in our list of regions seem to suggest a preferential activating function of these regions.

### Alu repeat enrichment in the vicinity of significant methylation changes and breakpoints

Recent studies (Witherspoon et al., 2009; Konkel and Batzer, 2010) showed that Short Interspersed Elements (SINE) and Long Interspersed Elements (LINE) could have a large impact on genome instability, increasing local recombination rates. Alu repeats are the most numerous transposable elements (one insertion every 3 kb) and Alu-mediated Non-Allelic Homologous Recombination (NAHR) are more frequent than other transposable element-mediated NAHR (Konkel and Batzer, 2010). Naturally, the question is whether the BEDMR loci that we identified can be linked to the presence of repeat elements such as Alu repeats.

**Figure 5:**
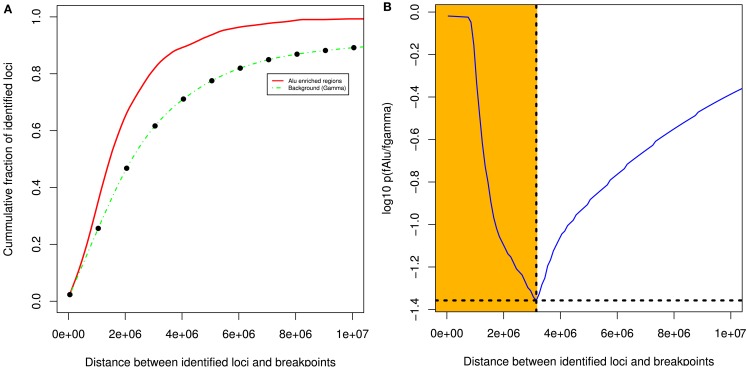
**Breakpoint enriched DMR tend to co-localize with Alu enriched regions**. The statistical evaluation shows that Alu enriched regions and BEDMRs co-localize within a distance of 3 MB **(A,B)**.

**Figure 6:**
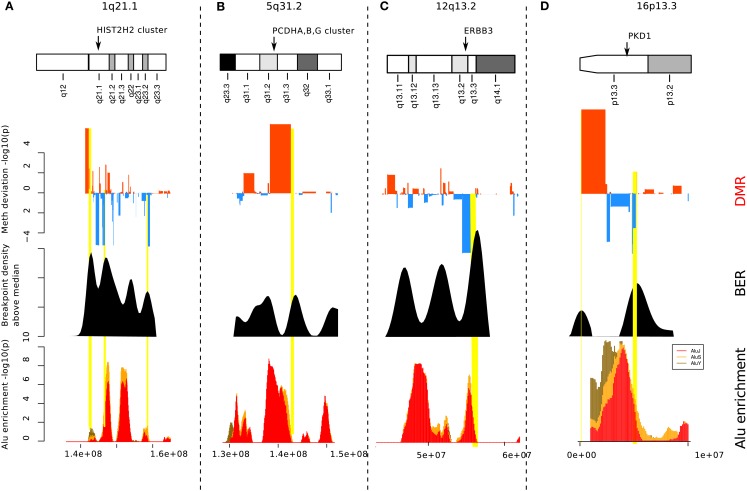
**Alu enrichment at BEDMRs**. Locations of significant DMRs (top track) near breakpoint enriched regions (middle track) are compared with local Alu repeat enrichment (bottom track). About 33 of the 93 identified BEDMRs overlap with an Alu enriched region. We show here four interesting regions on 1p21.1 **(A)**, 5q31.2-3 **(B)**, 8q24.3 **(C)**, and 16p13.3 **(D)**.

We observed significant association between Alu enriched regions and breakpoint dense regions where the repeat enrichment is localized and occurs within 3 Mb of BER (see Figure 5). Furthermore, when compared to the background, 33 out of 93 BEDMRs have significant Alu repeat enrichment (in 100 kb sliding windows, Wilcoxon test, FDR-corrected, *p* < 0.001). A large majority of them (24/33) are hypo-methylated compared to normal. A detailed diagram recapitulating methylation, breakpoint, and Alu repeat enrichment associations across 22 chromosomes is available in the Figure S11 in Supplementary Material. Some important chromosomal regions, chromosomes 1, 5, 12, 16 are presented in Figure 6. We found that loci with strong association between repeat enrichment and presence of a BEDMR pattern, affect important regulatory mechanisms in cancer. For example, the 1q21.1 locus (see Figure 6A) contains the HIST2H2, 2H3, 2H4 gene cluster, and the TSRC1, MCL1, ECM1 oncogenes. Hypomethylation of histone genes seems to be an important mechanism since we found hypo-methylated DMRs containing histone genes in 1q42.13 and 6p22.2. Deregulation of histone genes could contribute to genome instability in cancer by affecting chromatin structure. We also found again that the 5q31 protocadherin locus (see Figure 6B), is enriched with Alu repeats, upstream from the deletion locus. Interestingly, we found a hypo-methylated and Alu enriched BEDMR at the 12q13.2 locus containing ERBB3. This gene was found to be hypo-methylated in 45% of our samples suggesting a deregulation of this locus (see Figure 6C). Figure 6D presents, the 16p13.3 locus which includes PKD1 (associated with proliferation).

## Discussion

Our results provide evidence that there a statistically significant association between the locations of DMR (DMRs) and breakpoints enriched regions (BERs). In particular, 93 DMR regions occurred within a distance as short as 1 Mb from BERs, that we call breakpoint enriched DMRs (BEDMRs). It was interesting to see that DMRs were ubiquitous and were often hypo-methylated: 145 hypo-methylated and 72 hyper-methylated loci compared to the reference. This result is consistent with previous reported observations that global DNA hypomethylation is associated with punctual hyper-methylations in cancer genomes (tumor suppressor genes). Stratifying our analysis based on molecular subtypes (Luminal A and B, ERBB2+, basal-like), we found that the statistical association between DMRs and BERs was more significant than the expectation in each individual subtype [slightly stronger in Luminal B (73.5% of DMRs within 1 Mb distance of a BER), basal-like (65%), weaker in ERBB2 + (57.3%), Luminal A (55.5%), and all combined (42.9%), see Table S4 in Supplementary Material] and irrespective of which subtype was used (Figure S4 in Supplementary Material). More interestingly, we observed potential subtype specific BEDMR position patterns, see Figures S7–S10 in Supplementary Material for positional patterns of BEDMRs in Luminal A, Luminal B, basal-like and ERBB2 + subtypes and Figure S5 in Supplementary Material for a heat map recapitulating the different positional patterns. For example, 58 BEDMRs occurred only in basal-like samples, in particular in chromosome 6 and 18, which undergo frequent copy number alterations. However, these results may be further refined in another study with larger number of samples.

We focused on the regions where breakpoints and methylation pattern deviations co-localize. The analysis of a few important loci (Figures 4 and 6) allowed us to better understand the functional aspects of the BEDMR events. We detected changes across the entire chromosome, indicating that there was no positional preferences on the chromosome and possibly also no bias toward centromeres or telomeres. Subtelomeric regions are potentially unreliable for array based methods due to their highly repetitive DNA composition and high C + G content, and associated high methylation levels (Lee et al., 2009). Furthermore, BEDMR regions seem to occur in genic regions, in particular near genes with cancer related functions. In Figure 4, we showed a BEDMR at the 5q31.2 locus, which is epigenetically silenced in most cancers and contains the protocadherin A,B,G family, reported to be potential tumor suppressor genes modulating the canonical Wnt pathway in Wilms’ tumor (Dallosso et al., 2009) and other cancer related genes such as EGR1, CTNN1A, and CXXC5. We also found that BEDMR tend to deregulate genes involved in proliferation and invasion. For example, in chromosome 11, a BEDMR was found near the GAB2 gene that inhibits E-cadherin and promotes cell migration and invasion, in chromosome 1, the ADAMTS4 and PRDX6 genes were amplified and unmethylated in about half of the samples (Table S3 in Supplementary Material). Most interestingly, we found hypo-methylated BEDMRs and DMRs affecting histone gene clusters in chromosomes 1q21.2 and 6p22.1. The deregulation of members of histone gene family and chromatin remodeling factors such as the histone H3 demethylase JMJD1B may affect the chromatin structure and disrupt the coordinate signaling and repair, contributing to genome instability in cancer.

In the second part of our study, we investigated the relationship between the density of retro-transposable SINE elements (Alu) and genome instability. Observing the enrichment levels of the 93 BEDMRs, we found that in a significant fractions of cases (33/93), Alu repeat enrichment occurs in the vicinity of frequent recombination area. The role of Alu repeat elements in non-allelic homologous recombination events has been well described in the literature but many aspects are still unclear. Furthermore, the presence of SINE and LINE elements affects DNA methylation. It has been reported that the promoter regions of methylation resistant genes are twice as frequently enriched with SINEs and LINEs than the ones of methylation prone genes (Estécio et al., 2010). Furthermore, in a recent article (Li et al., 2012), it was shown that segments repeated in low-copy number regions (LCRs) were associated with genome instability and hypomethylation in the germline, and interestingly it was found that homebox, cadherin, and histone families were highly enriched in methylation deserts. In addition, a study on five cancer types using whole genome sequencing showed that transposable elements tend to occur in the vicinity of genes frequently mutated in cancer and biased toward regions of cancer-specific DNA hypomethylation (Lee et al., 2012). We found that 23 of the 32 BEDMRs enriched with Alu elements were hypo-methylated. We suggest that there might be a mechanistic relationship between hypomethylation, the presence of these repeat elements and genome instability, as also described in the literature. However, we recognize that further study is required to tease out how much these elements really contribute to the genome instability and whether the presence of oncogenes, change of methylation state, or local sequence repeat enrichment prevail in the mechanism.

Other studies have shown that fragile sites and associated genes are frequently deleted or rearranged in many cancer cells and have clearly demonstrated their importance in genome instability in cancer (Debacker and Kooy, 2007). Out of the 93 BEDMRs detected in breast tumor samples, 38 overlap with fragile sites while only 18 of them had an overlap with both Alu enriched regions and fragile sites (see Table S1 in Supplementary Material and associated genes in Table S3 in Supplementary Material). In these BEDMRs that overlap with Alu enriched regions and fragile sites, we found 35 genes with significant DNA methylation and copy number state in at least 20% of the patients. A remarkable locus is 1q21.3, overlapping with the fragile site FRA1A, in which SETDB1 and ARNT are amplified and unmethylated in 51% of the samples. SETDB1 is a histone methyltransferase and was previously shown to have oncogenic functions in melanoma, accelerating its formation (Ceol et al., 2011). Furthermore, ARNT is regulator involved in TF-miRNA feed-forward loop in cancer (Yan et al., 2012). Interestingly, the 1q21.3 locus has been also reported to be a melanoma susceptibility locus (Macgregor et al., 2011), suggesting that BEDMRs might target regions that are frequently fragilized or susceptible to deregulation in cancer. However, since approximately 30% of the genome is covered by fragile sites, it is unlikely that fragile sites contribute solely to genome instability and the presence of BEDMRs. The fraction of BEDMRs overlapping with fragile sites is indeed not statistically significant, suggesting that other elements contribute to the mechanism of association between BERs and DMRs. Furthermore, BEDMRs provide a much higher resolution insight into the relationship between breakpoints and differential methylation.

Our work focused on analyzing genome-wide patterns of DMR (DMRs) and (BERs) in relation to the genome architecture. Another important aspect relates to mosaicism. Breast cancer is a complex disease in which chromosomes are both affected in their structures and numbers, leading to mosaic karyotypes. At the resolution of our ROMA platform (ca. 40 kb), and using our breakpoint density functions we can appreciate and quantify regions of intense rearrangements made by the means of objective scoring schemes, as shown in Hicks et al. (2006) for firestorm indexes and Russnes et al. (2010) for WAAI and CAAI indexes of the Micma samples (also used in this paper). We should note that interphase FISH on 33 loci was used to confirm firestorms in a previous study, Hicks et al. (2006), 5 out of 12 validated loci described in the study overlapped with our DMRs and including one of these which co-localized with BEDMRs (11q14.1, Figure S12 in Supplementary Material). Our BEDMR regions can be seen as proxies to complex and frequent rearrangements. Nevertheless, one can also check the different clones of individual chromosomes using multi-color FISH. For example, in Bilal et al. (2012), a FISH experiment was performed on 36 samples which are ER + /HER2− of the Micma cohort to detect amplicons at 8q24.3, 8p11.2, 17q21.33-q25.1. These regions overlapped with BERs (8q11.2, 17q24.1-q25.1) and DMRs (8q24.3, 17q25.1) detected in our study (Table S1 in Supplementary Material). However true mosaicism can only be assessed in a future next-generation sequencing study of chromosomal translocations. Our assessment of co-localization of BERs and DMRs is a possible model toward genomic remodeling and temporal emergence of cancer.

Combining the different clues obtained throughout our work, we can sketch a tentative model that describes the relationship between the epigenetic and genetic changes in the genome associated with cancer and try to address the several questions that we asked in introduction. First, we showed that breakpoint occurrences seem to co-occur with local hypomethylation and BERs within 1 Mb. These regions, herein called BEDMR, were found in of presence of retro-transposable SINE Alu elements in 35% of these cases within a distance of 3 Mb. Second interrogation focused on the functional aspects of these structural and epigenetic changes and whether they had an impact on genomic regions which are prominent in cancer.

We found indeed that 8 of 93 BEDMRs were co-located with regions containing genes causally linked with cancer based on the Cancer Gene Sensus definition, but in fact this number could be larger since 47/93 of all BEDMRs and more interestingly 66% of the BEDMRs encompassed in a genic region (47/71) contained at least one gene reported previously in the literature as linked with cancer, e.g., PCDH family, SETDB1, ARNT, PRDX6, ADAMTS4, EGR1, CTNN1A, and genes involved in the chromatin structure such as histone gene families and remodeling factors. The number remains important even when taking a threshold of a minimum of three references for a gene (41/71, 57%). Our result was in agreement with other studies suggesting that transposable elements’ insertions, combined with abnormal hypomethylation and increased genome instability provide a selective advantage in tumorigenesis. Although no causal relationships can be inferred, we can say that each feature contributes partially to the preferential choice of certain loci for genome rearrangement.

## Conflict of Interest Statement

The authors declare that the research was conducted in the absence of any commercial or financial relationships that could be construed as a potential conflict of interest.

## Abbreviations

BEDMR, breakpoint enriched differentially methylated region; BER, breakpoint enriched region; CBS, circular binary segmentation; CGH, comparative genomic hybridization; CNV, copy number variation; DMR, differentially methylated region; EM, expectation-maximization; FDR, false discovery rate; MOMA, methylation oligonucleotide microarray analysis; ROMA, representational oligonucleotide microarray analysis.
